# Clinician Perceptions of a Novel Multicomponent Digital Care Assistant and Support Program for People After Stroke or Transient Ischemic Attack (CAPS) for the Secondary Prevention of Stroke: Qualitative Study

**DOI:** 10.2196/72873

**Published:** 2025-10-09

**Authors:** Liam Pearce Allan, Jane Li, Tara Purvis, David Silvera-Tawil, Jan Cameron, Marlien Varnfield, Vanessa Smallbon, Julia Bomke, Natasha A Lannin, Dominique A Cadilhac

**Affiliations:** 1 Department of Medicine, School of Clinical Sciences, Sub-faculty of Clinical and Molecular Medicine, Monash University, Level 2, Victorian Heart Hospital, 631 Blackburn Rd, Clayton, 3168, Australia, +61 75111865 ext 3; 2 Australian e-Health Research Centre, Commonwealth Scientific and Industrial Research Organisation, Brisbane, Australia; 3 Australian Centre for Heart Health, The Royal Melbourne Hospital, Melbourne, Australia; 4 Department of Neuroscience, School of Translational Medicine, Monash University, Melbourne, Australia; 5 Alfred Health, Melbourne, Australia; 6 Stroke Theme and Critical Care Research, Florey Institute of Neuroscience and Mental Health, University of Melbourne, Melbourne, Australia

**Keywords:** stroke, secondary prevention, mobile health, telemedicine, pilot projects

## Abstract

**Background:**

We co-designed the novel multicomponent CAPS (Care Assistant and support Program for people after Stroke or transient ischemic attack) to augment the secondary prevention of stroke.

**Objective:**

Following completion of a feasibility study, we sought feedback from Australian clinicians and service provider representatives (the potential deliverers of CAPS) regarding their perceptions of CAPS for secondary prevention and the pathways to real-world adoption.

**Methods:**

This was a qualitative descriptive study of clinicians and service provider representatives involved in the delivery of stroke care around Australia. A pragmatic convenience sample was obtained by contacting previous CAPS co-design study participants; leveraging professional networks (eg, LinkedIn); and distributing study flyers and newsletters via primary health care networks, general practitioner (GP) networks, and social media posts (Commonwealth Scientific and Industrial Research Organization’s LinkedIn pages). Semistructured interviews and focus groups were conducted virtually with clinicians and representatives of the Stroke Foundation (Australia). Qualitative content analysis was undertaken.

**Results:**

Overall, 18 clinicians and 3 Stroke Foundation representatives participated from 5 Australian states, including medical specialists, GPs, nurses, and allied health professionals. We collected their perceptions of CAPS, categorized as potential benefits of the program for secondary prevention, and the considerations for facilitators and challenges to real-world program implementation. The perceived benefits of supporting self-management for patients and facilitating informed decision-making for clinicians were identified. Discussions regarding program implementation included program initiation and duration, patient support considerations, and workflow alignment, which involved consideration of the barriers and enablers to uptake within primary care practice and Stroke Foundation outreach support programs.

**Conclusions:**

There was support from participants for the potential of CAPS to improve the secondary prevention of stroke. However, approaches for addressing the challenges raised by participants, including further implementation and integration considerations, such as sustainability of the model of care, are likely required for CAPS to be successfully embedded within clinical settings.

## Introduction

Globally, stroke is a leading cause of death and disability [1]. In Australia, a stroke occurs every 11 minutes, with over 400,000 people with a history of stroke now living in the community [2]. Those who survive their stroke are at high risk of a recurrent vascular event (13%‐40% cumulative risk at 10 years) [3,4], as are those who experience a transient ischemic attack (TIA) [5]. This risk can be reduced through the use of secondary prevention strategies [6]. It has been estimated that up to 80% of recurrent events could be prevented through uptake of healthy lifestyle behaviors (eg, exercise) in conjunction with the use of medication (eg, antihypertensives) [7]. However, the uptake and adherence to these prevention strategies remain suboptimal. Barriers cited by survivors include issues of access to services, lack of awareness or education following stroke, and psychosocial difficulties [8,9]. Further exacerbating the issue is the limited support provided to people following their stroke and discharge into the community [10].

Using digital health as a modality to deliver secondary prevention programs may be a method to improve long-term support and patient outcomes. Certain technologies (eg, smartphone apps) have been associated with improved management of chronic health conditions, including diabetes mellitus [11] and hypertension [12]. However, evidence for the use of digital health to improve the secondary prevention of stroke remains limited [13]. To ensure digital health programs are fit-for-purpose, end-user involvement in their design and development is crucial [14]. Engaging clinicians in this process ensures the resultant program is relevant to their work, aligns with their workflows, and may lead to better uptake in practice [15].

Consequently, we developed the novel CAPS (Care Assistant and support Program for people after Stroke or transient ischemic attack). The program was co-designed with people living with stroke or TIA and clinicians from different disciplines involved in delivering stroke care [16]. This iterative process included assessment of user needs from both patient and clinician perspectives, and user experience workshops. The resultant multicomponent program combines clinician-led, person-centered goal setting with the person living with stroke and facilitates risk factor monitoring through a remotely accessible clinician portal. Behavior change is supported through SMS text messages aligned to goals. Patient end users also access a purpose-built smartphone CAPS app and a commercial wearable device for tracking health-related measures.

The feasibility of CAPS was evaluated in a 12-week, single-group, open-label, pretest-posttest pilot study among 33 people after stroke or TIA [17]. The program was highly acceptable and satisfactory to people living with stroke or TIA, with evidence of potential improvements in cardiovascular and psychosocial health [17]. Participant feedback was generally focused on program modifications to improve usability.

In addition to seeking input from people living with stroke or TIA [17], it was important to collect feedback from clinicians and health service provider representatives as the potential deliverers of CAPS, if found to be effective. While patient experiences and perspectives regarding digital health are frequently explored, further work is required to understand clinicians’ perspectives and experiences [18]. Therefore, we sought to understand the perceptions of clinicians and service provider representatives regarding the overall program for the secondary prevention of stroke, as well as the potential pathways to the future adoption of CAPS into real-world use as a health promotion program. In this preimplementation study, we present the synthesis of the participants’ feedback to guide future development of CAPS and explore potential future models of care.

## Methods

### Study Design

A qualitative descriptive design was adopted, using semistructured interviews and focus groups. Methods and results are reported against the Consolidated Criteria for Reporting Qualitative Research (COREQ) (Checklist 1) [19]. This study was conducted following the feasibility study in which the digital health program was evaluated among people living with stroke or TIA (September 2022 to May 2023) [17]. A snowball recruitment process was used, where feedback from the first group of participants informed the selection of the appropriate cohort for the second group.

### Ethical Considerations

The interviews and focus groups conducted as part of this study were approved by the Monash University Human Research Ethics Committee (30521) and the Commonwealth Scientific and Industrial Research Organization (CSIRO) Health and Medical Human Research Ethics Committee (2021_118_RR). Consent was collected electronically prior to commencement. All data were stored securely according to institutional guidelines. Participants were provided a gift card to acknowledge their contribution; amounts were recommended by our institution and stakeholder advisory groups. To maintain privacy, participants were given pseudonyms for data analysis and reporting.

### CAPS Components

**Figure 1.**
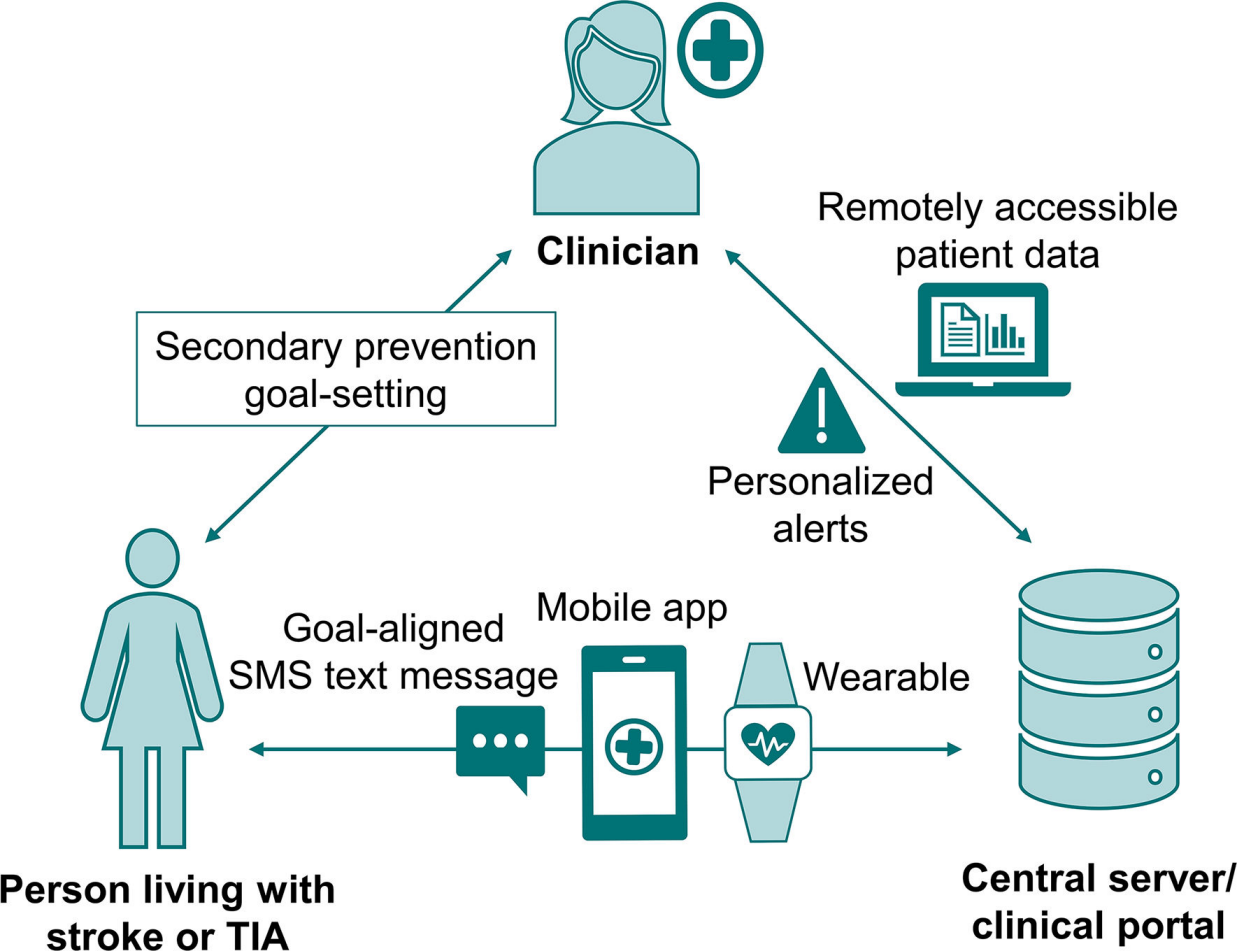
Components of CAPS (Care Assistant and support Program for people after Stroke or transient ischemic attack). TIA: transient ischemic attack.

The CAPS components are described in detail in previous publications [16,17]. In brief, participants used a standardized process to set 1-2 secondary prevention and person-centered goals using the SMART (specific, measurable, achievable, realistic, and timebound) format [20]. The template and procedures for this process were adapted from a phase III randomized controlled trial (RCT), which involved using SMS text messages to support goal attainment across the domains of secondary prevention, activities of daily living, self-management, psychosocial health, and accessing health care [21,22]. The clinician-facing technology consisted of a remotely accessible web-based portal (Figure 1). Patient profiles were created and tailored using personalized health measurements and alert thresholds, upload of SMART goals, activation of SMS text messages aligned to goals adapted from the above study, and education links. Alert messages within patient profiles were activated for measures outside thresholds sourced from clinical guidelines (eg, systolic blood pressure >140 mmHg). This process also included an automated email to the monitoring coordinator and SMS text messages to the participant. Patient-facing technology consisted of 3 components. The first was motivational goal-aligned SMS text messages, sent at a rate of 2 messages per goal per week, with some containing educational hyperlinks. The second was the purpose-built smartphone CAPStroke app, where patients entered health data daily (with different measures selected through the portal) and could review their health data, enter journal notes (audio, text, and images), activate medication reminders, and access education resources. The third was a Bluetooth-synchronized smartwatch or fitness tracker that passively collected patient data with automatic upload to the portal and app.

### Participants

A pragmatic convenience sample of different clinicians involved in the delivery of stroke care from various parts of Australia was invited to participate in this qualitative study. Several recruitment strategies were used. For the first group, we sent invitations to participants who were involved in the co-design of CAPS and had expressed a willingness to contribute further to this research, and also reached out to our existing professional networks (eg, LinkedIn) between May and June 2023. For the second group, various methods were used to recruit stakeholders who were identified by participants in the first group as important in providing or responding to features of CAPS. These included general practitioners (GPs) and representatives of the Stroke Foundation (Australia), who provided in-reach support services to people living with stroke, including advice on secondary prevention. The recruitment strategies for the second group included distributing study flyers, internal organization newsletters, and personal invitations over a 4-month period (January to April 2024) via primary health care networks (PHNs) (Tasmania & Gippsland), Monash Health GP Liaison Unit, Monash University Practice-based Research and Education Network (MonREN), and GoldNet Research. A marketing post was also made on CSIRO’s LinkedIn pages targeting GPs, and individual GP clinics were contacted via email or directly through their website.

### Data Collection

Online semistructured interviews and focus groups were conducted separately with clinicians and service provider representatives based on their availability and preference to meet. During semistructured interviews and focus group sessions, participants were provided with a background of the purpose of the study and then an overview of the program, including video demonstrations of the CAPStroke smartphone app and clinical portal. Following this, participants were asked a series of open-ended questions regarding their perceptions of using CAPS for the secondary prevention of stroke and the potential adoption of CAPS into real-world use. The questions posed within the semistructured interview guide were adapted from previous work and subsequently refined with input from the research team. This was to ensure that all important concepts, specific to the project objective, were captured (Multimedia Appendix 1) [23,24].

For the first group, sessions were conducted remotely via the Cisco Webex video conferencing system (May and June 2023). Four researchers were involved in facilitating the sessions, with 2 researchers present in each (JC: female, PhD, clinician researcher; DS-T: male, PhD, digital health researcher; JL: female, PhD, digital health researcher; LPA: male, BSc, PhD candidate). For the second group, the sessions were conducted remotely via Microsoft Teams video conferencing (March and June 2024). Two researchers were involved in facilitating the sessions, one of whom was involved with the first group (TP: female, PhD, clinician researcher; JL). Some researchers were known to some participants from the co-design process or from professional relationships.

Sessions lasted approximately 60 minutes and were audio-video recorded with consent, using Webex or Microsoft Teams. Notes were taken by the researchers during each session. Audio recordings were professionally transcribed verbatim.

Rather than basing the sample size on data saturation, in this study, we focused on achieving adequate information power by using purposive sampling, having clear objectives, and obtaining rich, detailed interview data [25]. According to this framework, the more relevant the cohort is to the study aim and the more specific the analysis, the fewer participants are needed.

### Data Analysis

To minimize potential bias, a semistructured interview guide was used, and coding was completed by 2 researchers (JL and LPA) who did not have prior relationships with the participants. Qualitative content analysis was undertaken by 2 researchers (JL and LPA), using NVivo software (QSR International) [26]. As this was a preimplementation study in which potential modifications to the program and future models of care were explored, this approach was used to systematically organize the data and generate practical insights [26]. Analysis of the first group occurred prior to recruitment of the second group, with all data combined. Preliminary initial codes were established based on the interview questions and feedback received during sessions using an integrated approach, which involved applying deductively derived codes based on the interview questions with inductively derived codes from session transcripts [27]. Coding was cross-checked and discussed between the 2 researchers prior to the second round of the coding process and writing of the results. For the second group, additional steps included discussing and reviewing initial notes taken during the interviews and focus groups among 3 researchers (TP, JL, and LPA). The categories and subcategories generated in the second group were reviewed by a third researcher (TP) and visualized using Miro software (RealtimeBoard, Inc). Coding was cross-checked and discussed among the 3 researchers prior to the second round of the coding process and writing of the results. Final coding for both groups was discussed with 2 additional authors (JC and DS-T), following the writing of the results. Disagreements were resolved through consensus.

## Results

### Participant Characteristics

**Figure 2.**
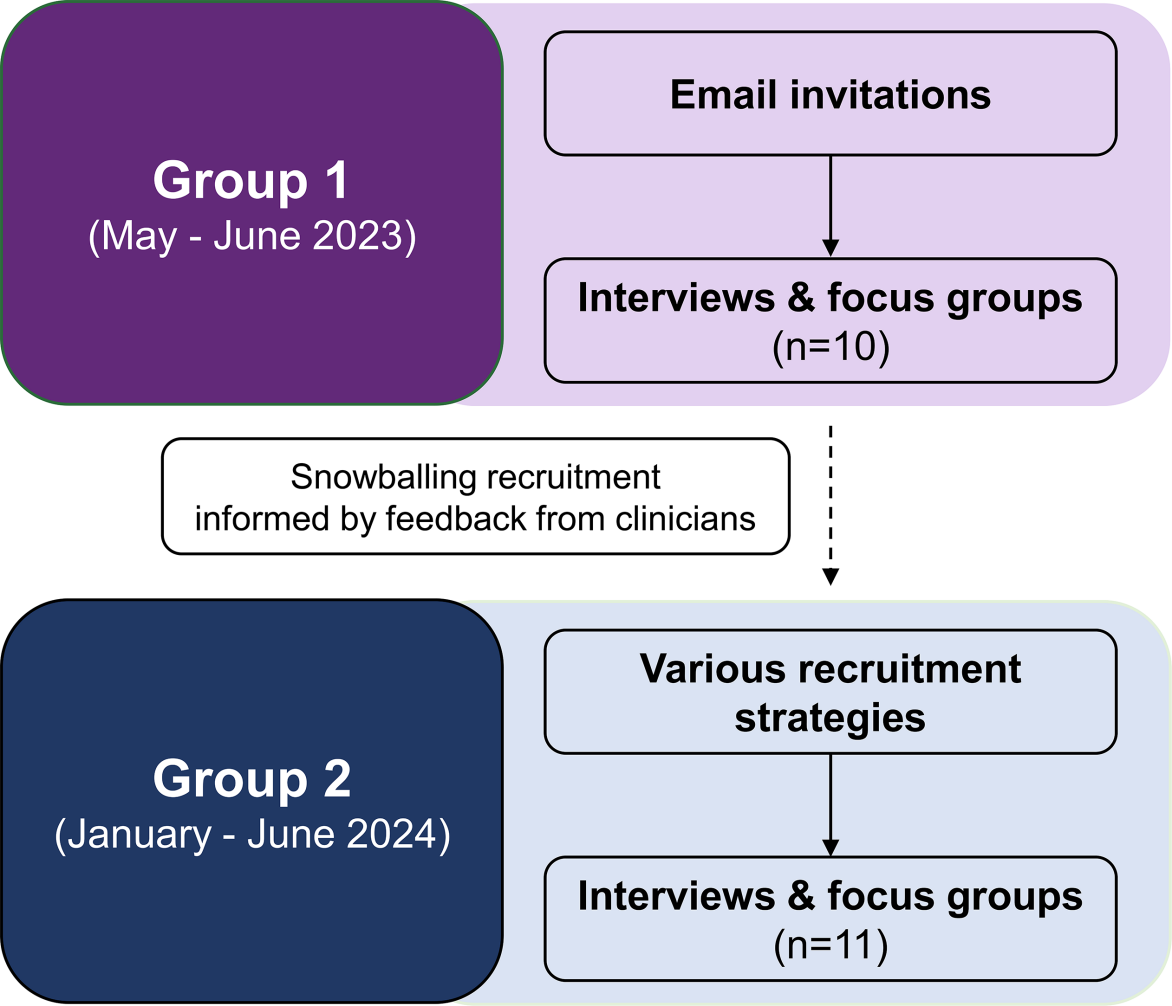
Flow of participant recruitment for the first and second groups of the study.

Eighteen clinicians and 3 Stroke Foundation representatives participated (Table 1). Eleven participants attended one of the focus groups, ranging from 2 to 3 participants per session. Ten participants had one-on-one interviews. The participant flowchart is provided in Figure 2. Clinicians had various clinical backgrounds, including specialists, GPs, nurses, and allied health professionals. Clinician experience spanned the stroke continuum of care. They were working in various settings, including acute care (n=4), rehabilitation (n=3), and primary practice (n=9). One clinician also worked in community-based care as an adjunct to their acute role. Stroke Foundation representatives worked across various primary and secondary prevention services offered by the provider. All 3 representatives also had clinical backgrounds (nursing and allied health).

**Table 1.** Participant characteristics.

Characteristic			Value (N=21), n (%)
Female sex			11 (52)
Australian state of residence			
New South Wales			5 (24)
Queensland			4 (22)
South Australia			2 (10)
Tasmania			1 (5)
Victoria			9 (43)
Profession			
General practitioner			9 (43)
Medical specialist			
Neurologist			2 (10)
Stroke consultant			1 (5)
Nurse			
Registered nurse			2 (10)
Nurse practitioner			1 (5)
Allied health professional			
Occupational therapist			1 (5)
Physiotherapist			1 (5)
Speech pathologist			1 (5)
Stroke Foundation representative^a^			3 (14)

a Nurse, physiotherapist, and exercise physiologist.

### Categories and Subcategories

We present the results and subcategories related to the categories of “*perceived benefits*” *of CAPS for secondary prevention* and *considerations for real-world* “*program implementation*,” including facilitators and barriers to future program uptake (Table 2).

**Table 2.** Summary of categories and subcategories.

Major category	Subcategories
Perceived benefits	Overall perceptions of the program and technology Benefits to the patient - Supporting patient self-management and accountability - Appropriateness of the program technology Benefits to the clinician - Facilitating informed decision-making
Program implementation	Program initiation and duration Patient support considerations - Encouraging patient ownership and accountability - Barriers to patient engagement Workflow alignment within existing programs and models of care - Alignment with Stroke Foundation services - Alignment with primary practice Practical factors affecting program delivery - Barriers to uptake in primary practice - Reimbursement and funding considerations An independent model of care

#### Perceived Benefits

In this section, we describe the perceptions regarding the overall program and the potential benefits to the patient and the clinician.

##### Overall Perceptions of the Program and Technology

The majority of clinicians saw the value of CAPS as a “comprehensive” program that “integrates” multiple functions for health monitoring and tracking. They also praised the program as a novel product “a few steps above what is currently available in the market” for people living with stroke or TIA in the community. Many clinicians indicated that the technological components appeared to be “very user-friendly” and “well-designed,” making them easy to navigate.

*I’m part of a clinical entrepreneur program at the moment and […] there’s so much happening in this space. I certainly haven’t seen anything similar to this around secondary prevention of stroke and TIA.*

[Participant #6, GP]

Some GPs also raised the wider applicability and potential use of CAPS in the primary prevention of stroke and across different chronic conditions, given that the risk factors for stroke prevention are relevant to many other disease states and health conditions.

*It’s almost like it could just be a generic patient thing, not even condition specific. And if you had enough individualized goals, I mean I think it could be nearly for everyone.*

[Participant #18, GP]

##### Benefits to the Patient

###### Supporting Patient Self-Management and Accountability

The potential benefits of CAPS as an option for improving self-management and “optimizing” the secondary prevention of stroke through its emphasis on self-management were raised. Clinicians indicated that the program could be used to enhance postdischarge care, providing clear instructions, support, and information to patients in a “straightforward” way. This was seen as preferable to overwhelming them at a time when they are often feeling “lost” or vulnerable after discharge from an acute hospital. Participants saw CAPS as useful for encouraging patient self-management, with the data collection serving as a motivational tool to support accountability to themselves and to their health care provider. A GP suggested the program could provide a safety net, particularly for patients who might be deterred from presenting to their GP, and could help “pick up” patients with worsening health.

*[CAPS could help] in one or two ways, [for the patient that has] kind of completely changed their ways and are highly motivated […] this type of program no doubt would facilitate that [self-management] for them. And then some patients obviously spiral the other way and get quite depressed and, you know, fall into a bit of a hole so, but this, you know, this might be useful to give them something tangible to hold on to.*

[Participant #12, GP]

*[…] to have this recommended, just so [patients] don’t feel like they’ve just literally been kicked out the door and that their stroke didn’t matter, I think it will help them to accept some of that and process some of those changes and actually set up good life skills going forward. I think it’s - it just literally would be a safety blanket for them, but a positive safety blanket.*

[Participant #1, registered nurse]

###### Appropriateness of the Program Technology

Feedback regarding the patient-facing components of the program indicated that the measures collected through the app from the daily check-in were “all valid” for patient management. However, a few of the specialists (a neurologist and stroke consultant) noted that including subjective measures, such as mood or distress, was less important for the secondary prevention of stroke and that objective physiological measurements, such as blood pressure, should be the main focus of the program, suggesting the daily check-in to be “pared back.” Conversely, GPs stated that while those physiological parameters were very important, subjective psychosocial measures were also important in long-term patient management and well-being.

*I think a lot of patients don’t realize once you’ve had a stroke, there’s a higher chance you’ll be depressed, so I think it’s good for them to be thinking about it, so maybe they can be more proactive about it as well.*

[Participant #18, GP]

Clinicians also recommended tailoring the frequency of each measure for patients (rather than being exclusively daily) and taking advantage of the electrocardiogram features of some of the wearable devices for monitoring atrial fibrillation.

*[smartwatches having] the capacity to do an ECG […] that’s going to completely transform diagnosis of AF into the future.*

[Participant #6, GP]

### Benefits to the Clinician

The benefits of using CAPS for the clinician were discussed less often, with a few of the clinicians stating that the program would have more benefits for the patient than it would for the clinician.

#### Facilitating Informed Decision-Making

Most of the GPs mentioned that they could leverage the rich data provided by CAPS to improve patient care and better target the risk factors of concern. This included enhanced monitoring of psychosocial well-being, highlighted as an important factor in improving patient treatment “outcomes and compliance*.*” Clinicians emphasized the value of accessing longitudinal data that can offer greater clinical insights than the limited data that might be collected in a single consultation (eg, blood pressure readings). One GP also indicated that the potential of improved patient outcomes would be motivating for clinicians to use the program for patient management.

*I guess the things that I would find beneficial is just having more data […] having more blood pressure readings, having more information […] if they come in and they’ve got all this information just available for us, we can make much more informed decisions much quicker.*

[Participant #18, GP]

The importance of presenting the data in an accessible way in the clinical portal was raised, for example, using a traffic light or matrix system to easily identify data outside of clinically recommended parameters (eg, hypertension and hypercholesterolemia). By doing so, the clinician could “very quickly” get an idea of the current health needs of the patient and use the “tangible” data to target areas of improvement (eg, blood pressure).

*[…] I’d love to just be able to type in total LDL [low-density lipoprotein], HDL [high-density lipoprotein] ratio, triglycerides and then they can just sort of see […] how well are [they] in the green?*

[Participant #10, nurse practitioner]

*I don’t want a data kind of avalanche coming our way, it would be more tangible bits of information that we can move the needle with.*

[Participant #12, GP]

### Program Implementation

A major focus of the focus groups and interviews was to understand the potential adoption of CAPS into real-world use. In this section, we present themes related to the optimal time for delivering such a program, patient-level considerations, and the identification of potential models of care. We also explore the facilitators and perceived barriers to program uptake at the clinician level.

#### Program Initiation and Duration

Clinician sentiments for a suitable program initiation time after stroke were varied, ranging from immediately upon discharge to slightly longer follow-up periods (ie, a few weeks to a few months after discharge). However, the general consensus was that patients would receive the most benefit from the program if delivered within 3 months of discharge from the hospital. This aligned with feedback provided by patient participants [17].

When queried, nearly half the clinicians noted that the program should be delivered upon or soon after discharge, particularly after having been seen by specialists such as neurologists and cardiologists. They felt this was an appropriate time given that patients and their family members are “quite motivated” to implement changes and are more “susceptible to behavior change” and to learning healthy “habits.” This could be done by introducing CAPS as part of the Stroke Foundation’s MyStrokeJourney, a resource provided to patients to support the “transition from hospital to home” [28].

*CAPS really has value [delivered] very acutely after an incident, when patients are more likely to engage.*

[Participant #14, GP]

However, the other half of the clinicians felt it would be more appropriate to introduce CAPS a few weeks to within 3 months after discharge, with 3 elaborating that waiting a period of time after discharge was ideal. This is because patients are often overwhelmed and need further information and support at this time.

*[…] we do a three-month follow-up in all the strokes […] that to me that would be the sweetest spot to introduce this […] they are overwhelmed, there are multiple competing demands, multiple things to still get their head around. Three months the patient already comes with a list of questions, and I see that as a nice time.*

[Participant #4, neurologist]

A more flexible model of care was also suggested by a few of the clinicians, with the possibility of introducing the program at any time to patients who were discharged directly from hospitals and who have been unable to access other support services. They also felt it would be appropriate to offer the program to patients who were not given enough education about the secondary prevention of stroke at the time of their event, as identified by their GP.

*It really depends doesn’t it, if it’s a TIA, they’ve had a fright and they’re very motivated to stop that from ever happening again or stop them from ever having a stroke. If they’ve had a major stroke then […] they’ve got a whole grief process and a rehab process, so I think it needs to be individualized […].*

[Participant #6, GP]

#### Patient Support Considerations

##### Encouraging Patient Ownership and Accountability

Clinicians expressed clearly that the onus should fall on the patient to self-manage for secondary prevention, rather than assuming and relying on the oversight of clinicians, who are likely to be monitoring the data sporadically. This entails patients being “empowered” to take “ownership” of their health and being “confident” in knowing when to seek help from their primary carer. However, several GPs raised concerns about patients interpreting their clinical information without the support of a clinician, feeling it “opened up a can of worms.”

*[…] there has to be an expectation put on the patients around how much responsibility they take for their health; you know they can’t completely load that back on the clinicians.*

[Participant #12, GP]

*I think […] most people are trying to take more responsibility for their health and something like this could definitely help them along the way. And I think allows them to […] take that ownership. I would think they would enjoy it.*

[Participant #5, occupational therapist]

##### Barriers to Patient Engagement

Clinicians raised some challenges patients could experience with accessing and using the program. There were concerns that some people with stroke would be unable to access the program due to not owning the right technology and that some elderly patients may lack the technological literacy for the program. Additionally, the potential difficulties in providing equitable access to the program were emphasized, particularly in culturally and linguistically diverse communities, including Aboriginal and Torres Strait Islander people. However, it was raised by 3 clinicians that patients could be supported and trained to use the technology relatively easily.

*Of course, there is going to be a proportion of patients who can’t manage a smart phone, don’t speak English, don’t know their Apple ID [identification] or password. Those sorts of things are a challenge and those people are already the least well served of our population, particularly people who don’t speak English.*

[Participant #10, nurse practitioner]

*I think some people might be a little IT [information technology] illiterate, but […] I wouldn’t say that you couldn’t teach someone how to do that pretty easily.*

[Participant #18, GP]

Some of the clinicians noted that CAPS would predominantly benefit motivated patients, who would find the program “exceptionally helpful,” but that less-motivated patients may struggle to engage. Similarly, there were discussions about the difficulty of patients to adhere to programs, with barriers to engagement, such as fatigue or depression, as well as previous challenges with requesting patients to complete data input for extended time periods.

*[…] those people who are motivated will probably engage in this and those who probably need it are maybe less inclined to engage for reasons. That’s always a battle.*

[Participant #16, GP]

### Workflow Alignment Within Existing Programs and Models of Care

Clinician opinions regarding who could best deliver the program varied. In the first group, most clinicians recommended a GP-shared model of care, feeling the program aligned with the purpose of primary practice. Most clinicians highlighted the benefits of including multidisciplinary care professionals (nurses and allied health professionals) for supporting secondary prevention. Given the current service provision by the Stroke Foundation, the alignment of CAPS with their existing programs was considered. In the second group, perceptions of the GPs regarding the potential model of care and workflow alignment varied, and several challenges to the program being GP-led were raised. This concept was explored with GPs, and further information about their perceptions is provided below.

#### Alignment With Stroke Foundation Services

Some of the services provided by the Stroke Foundation, relevant to CAPS, include the Living Well After Stroke program and the Stroke Navigator service. The first is a secondary prevention program delivered via telehealth to improve self-management and education for people after stroke. The Stroke Foundation representatives indicated that no objective measures (eg, blood pressure) are taken by the Living Well After Stroke program, with more focus on supporting participants to achieve their program goals and equipping them with a toolkit for self-management. In the second service, the Stroke Navigator, patients are contacted by staff following discharge from the hospital to support the access of various services for managing recovery and secondary prevention. Given this, the representatives were asked if they felt the Stroke Foundation could and would include CAPS as a service in their current model. The Stroke Foundation representatives indicated that while CAPS aligned with Living Well After Stroke and the purpose of foundation activities, there was currently no bandwidth for it to be incorporated as a Stroke Foundation–led service. However, 2 representatives felt that the Stroke Navigator service could be used as a referral or registration pathway for people living with stroke returning to life in the community. When queried regarding the placement of the program, 2 of the 3 representatives indicated the importance of partnering with GPs to deliver CAPS.

*[…] that’s their key, that’s where their medical care, their recovery is mainly managed […] in general practice.*

[Participant, Stroke Foundation representative]

#### Alignment With Primary Practice

Most GPs pointed out that CAPS aligned well with GP Management Plans currently used to facilitate chronic disease management (CDM) in primary practice [29]. These plans are described in further detail in the subsection *Reimbursement and Funding Considerations*. It was raised by 1 GP that frequent touchpoints with clinicians, as required in CAPS, which could be facilitated by aligning with these care plans, are highly beneficial for patients in programs such as these and can support continued “motivation” and “achieving goals.”

*[...] a lot of this is based on personalized goals... and this is how this could be integrated into...I could see how this could make those plans just amazing...and this program sort of goes for 12 weeks, and I was just thinking that would fit with those GP management plans so much better. And the goals that are in the app are almost exactly the goals that I write for my patients now […] adding more work is often not encouraged by GPs, but I think how this could integrate into general practice, that would be really useful.*

[Participant #18, GP]

There was variability reported in the perceptions of who would potentially be involved in the various tasks of onboarding and delivering CAPS. Some GPs felt that practice nurses were best equipped to onboard participants into the program, including familiarizing them with the various technologies. It was also mentioned that nurses could support patients to set secondary prevention goals. One GP stated a preference for their practice nurse onboarding patients but they themselves [the GP] setting goals with the patient. Another GP noted that either the GP or a nurse could onboard patients, depending on the practice (eg, GPs in small practices without a nurse). One GP also raised the possibility of referring patients to an external service for registration and onboarding, and then having an “agreed shared care” model with the external entity.

*I could see how a practice nurse involved in this could be really helpful, because they have a little bit more flexibility on time […] getting the nurses to set up this with the patient would […] take that pressure off the GP doing it.*

[Participant #18, GP]

GP opinions regarding who was best equipped to monitor the data were split between GPs and practice nurses. Some indicated that both were potentially viable options in the interviews. Those of the opinion that it should rest with GPs felt that it should be their responsibility as they are the primary point of “medical care” and are managers of “recovery” for patients after stroke.

*They’d [GPs] definitely be able to do the monitoring side of things.*

[Participant #18, GP]

Others noted that the practice nurses had the “skill set” and more availability to monitor patients and would be able to “spend more time” than the doctors supporting patients. It was also mentioned that the nurses could then act as a “filter” or could triage patients and “flag” particular concerns with the GP when patients attended appointments.

*[…] we do health assessments, data gathering, practice nurses are fully trained to do that in our clinic. We do care plans, […] falls assessments and so forth, things that take time and I don’t have that. My practice nurses do it on my behalf.*

[Participant #13, GP]

### Practical Factors Affecting Program Delivery

#### Barriers to Uptake in Primary Practice

A number of challenges to embedding CAPS in primary practice were raised.

The majority of GPs highlighted the lack of time they currently have available with an “insurmountable” workload, without the addition of a “high touch” monitoring program. They expressed concerns about the increased workload, potential for missed alerts, and legal implications of managing the additional information, especially outside regular hours. Additionally, 1 clinician raised the lack of support for GPs, particularly those in rural locations.

*[…] there’s a whole other workload attached to GP workload with no more remuneration or care because if the public system is suffering, then it’s, we have this finite plate of things we have to do and people just keep adding, adding, adding, there’s CAPS, there’s obstetrics, there’s psych, there’s – but the job is still, you know 40 hours a week, so unfortunately some things are going to fall off the plate if you keep adding without creating a new job or adding more capacity or hiring new GP’s or nurses.*

[Participant #17, GP]

Some of the GPs raised issues with timeliness in responding to alerts activated within the portal when patient measurements are outside guideline-based thresholds. This included consideration of alerts being activated during periods where GPs are away, “out of hours,” or not accessing the information until an extended time afterwards. One GP shared a method for addressing this current issue in their practice, where out-of-hour patient telephone calls are forwarded to after-hours medical clinics, allowing patients to still be treated. However, this comes with the caveat that the after-hours clinics cannot access clinical information from the GP and vice versa.

*[…] they sort of just get redirected to after-hours […] if they need to see a doctor then as well. There’s pretty much no access to our notes, like those afterhours doctors can’t see our file at all.*

[Participant #18, GP]

Most of the GPs interviewed and one other clinician (registered nurse) had concerns regarding the duty of care and medico-legal responsibility associated with the expectation from patients that there is a clinician responding to alerts in a timely and appropriate manner. An example situation provided was an alert being activated while a GP is on leave or “out of hours,” which does not get actioned and leads to a medical emergency, and the liability of the GP in that situation. Several GPs felt that adopting the program could lead to increased insurance premiums. These legal and cost implications were highlighted as deterrents to the uptake of the program.

*I think in terms of, from the medical legal point of view, it opens up conditions to being liable for clinical information that comes across your desk, because if you see something that’s abnormal and you’re on leave or you’re away and it doesn’t get actioned and then the patient subsequently has a stroke or something like that then who is at fault?*

[Participant #17, GP]

Interoperability with the current medical software was raised as critical in the adoption of CAPS by most of the GPs interviewed and another clinician (nurse practitioner). Two GPs raised the challenge of using an additional interface in the time-poor environment. To access and use the information collected in CAPS, the GPs stated that the data would need to be automatically “integrated” into the patient’s medical file in the practice software. According to them, this could include “populating” the patient file in the practice management software or providing “downloadable data.” One GP felt that being able to integrate with the practice software would be “game-changing.” Another suggestion was to include a link to the CAPS clinician portal within the patient profile in the practice software for seamless access.

*[…] as a GP I want it all in the one thing, I want it all in best practice, I want it all in medical director.*

[Participant #18, GP]

*I think as soon as you’re asking GPs to go outside their medical software and log on to another web page, they’re not going.*

[Participant #12, GP]

Another concern raised by a few of the GPs was the management and storage of patient data on an external platform. Citing “privacy issues,” they flagged that GPs often worry about sending data to third parties and desire clear “labeling” of how their patient data are being stored and used. One GP indicated that backing from a trusted organization, for example, data being hosted and protected by the CSIRO (a federal government research organization) or elsewhere by the government, would help allay those concerns.

Some GPs highlighted cost and reimbursement as major barriers to implementing the program in primary practice. This is explored further below.

#### Reimbursement and Funding Considerations

Aligning consultation and management of patients under the Medicare Benefits Scheme (Medicare), Australia’s universal health care insurance program, was suggested by most GPs. This could be done by aligning the program delivery (including onboarding) and associated consultations under the CDM services provided by the GP. CDM services were introduced to provide further Medicare subsidized support to patients with chronic medical conditions [29]. By doing so, the nurses involved in supporting the delivery of CAPS could be reimbursed via existing Medicare rebates within the management plan codes for their time and consultations, which was highlighted as vital with the “razor-thin” margins of a GP practice.

*[…] you can probably fall within the same consult as the GP management plan, like a chronic disease management plan, because that also requires goal setting to do it correctly.*

[Participant #14, GP]

Similarly, some GPs indicated they would privately bill the patients, referencing the “undercurrent” in the general practice of continued reductions in bulk billing (where Medicare benefits fully subsidize health services).

*[…] if we created a job and we paid someone to be monitoring it possibly, but there would be, it would be a paid service and the patient would have to foot the bill for it.*

[Participant #17, GP]

Another GP raised the possibility of using private health insurance to cover the sessions, given that the insurers are “incentivized” to reduce members using health services and having recurrent events. This could be used to then incorporate health coaches into the program to support and guide the patients and take the burden of monitoring from GPs. However, they acknowledged that this could exclude patients who do not have private health insurance.

*[…] the private health insurers can pay the health coaches to actually help patients through this journey and […] the app can actually be part of the private health insurer hospital prevention program to say, a tool that health coaches can use […] set goals for [the patient] and the alerts will streamline where the alerts go, or will help [the patient] with when is it relevant to see a GP.*

[Participant #14, GP]

### An Independent Model of Care

An alternative proposal by several GPs was that the program could be run independently as a standalone private service, providing a solution to the current barriers GPs face. One GP mentioned that this could lead to more “feasible,” “efficient,” and cost-effective care by training independent clinical staff to solely onboard, monitor, and respond to data. These staff could then link in with GP services to share the information collected. Another GP highlighted how previous programs supported by a PHN have worked, for example, having independent clinical staff travel to different clinics, provide heart health checks, and then feed that information back to the GP.

## Discussion

### Principal Findings

We have described the perceptions of Australian clinicians and Stroke Foundation representatives regarding CAPS. This included their opinions regarding the potential perceived benefits, and the facilitators and challenges to the future adoption of this novel digital program as a health promotion program delivered in the community. We report that clinicians from different disciplines and the continuum of stroke care saw the value of a digitally delivered multicomponent secondary prevention program, which was discussed as a potential model of care. We present their insights into the challenges of integrating these types of programs into primary practice.

Both the clinicians interviewed in this study and the end-user participants living with stroke or TIA in the feasibility study felt the program could provide essential support if delivered shortly after discharge [17]. While secondary prevention begins in the hospital with medications, care plans, and risk factor education, not all patients receive these. In 2023, 30% of patients discharged from Australian hospitals did not receive a care plan, and 28% were not educated regarding risk factor management [30]. The transition from hospital to home is often poorly supported, leading to unmet needs, particularly in healthy lifestyles and uptake of secondary prevention [31]. The lack of support provided may contribute to many people living with stroke reporting long-term unmet needs, particularly in the areas of healthy lifestyles and secondary prevention [32,33]. CAPS could be used to provide essential information for self-management and ensure patients feel better supported after discharge [34].

Clinicians identified issues of accessibility, particularly “digital exclusion”, as a potential barrier to patient uptake of the program. Digital access is now a key social determinant of health [35], which can be affected by factors such as digital literacy, age, education, and cultural diversity [36,37]. People with lower digital literacy, including those from culturally diverse backgrounds, are less likely to access and use digital technologies for their care [38]. To bridge the digital divide, interventions should be made accessible through various methods, such as using simple language, offering alternative formats (eg, audio or icons rather than text) [38,39], providing devices [13], co-designing interventions to ensure they are fit-for-purpose [13,38], and offering individual training in the use of technology [40]. In the CAPS feasibility study, training was provided via an image-based step-by-step manual, a tutorial video, and a telephone call. This may have contributed to the high level of participant satisfaction with the usability of the smartphone app and wearable device [17].

An additional barrier identified was the challenge of engaging patients who lack motivation to improve their health. While we observed sustained technology usage in the feasibility study [17], that finding was among people who chose to participate in digital health research. The broader stroke population may be less incentivized to engage with a digital health program and less motivated to improve their health. However, previous studies have found that particular features, such as the ability to review the data and active participation in health management, can improve motivation and engagement [41-43]. Person-centered goal-setting also increases motivation among people living with stroke [44]. While these features are part of CAPS, the program needs further evaluation in a larger, more representative population to assess motivation and adherence.

Several barriers to the adoption of a digital health program into clinical practice were identified. Stroke Foundation representatives noted limited availability for integration with existing services in their stroke-specific models. In particular, barriers of adoption into primary practice were discussed. Scarcity of resources was a major concern of the clinicians interviewed, with limited time and personnel to regularly monitor and respond to patient data. Previous studies have also shown that mobile health technology (eg, smartphone apps), telemonitoring, and self-management tools can increase clinician workload [45], hindering the implementation of evidence-based stroke care guidelines [46]. Digital health programs should support, not disrupt, workflows, and clinicians must be involved in their development to ensure they are effective and fit-for-purpose for both patients and clinicians [47].

The first group of clinicians noted that GPs were the best placed health care providers to deliver a program such as CAPS. However, the GPs cited a number of barriers, as explored above, that raise challenges in delivering CAPS in the current primary practice setting. As such, a GP-led model of care may not be feasible at scale. Involving nurses (for example, the specialist nurses in the New South Wales Chronic Disease Management Program) [48] to support a shared model of care may be more plausible. Previous research has indicated that nurse-led remote patient monitoring is feasible and has positive clinical outcomes [49].

With training, a nurse could develop patient-centered goals, monitor and respond to data, and provide action points or feed important information back to primary care. This also leverages an existing workforce that is trained in health promotion, CDM, and patient monitoring. As suggested, delivering CAPS as an independent nurse-led service, where clinical summaries are securely fed back to primary practice, could alleviate some challenges of integration with the various practice management software. Given the fragmentation of management software used across Australia, integrating CAPS into each would be technically and financially challenging. Data sharing through secure formats (eg, HL7 or encrypted PDF reports) to primary practice could be a more practical approach.

Consideration of the sustainable implementation and delivery costs of CAPS is vital, given that they are key barriers to the uptake of digital health programs [50]. While aligning the program under the Australian Medicare–subsidized CDM plan as a potential reimbursement model was discussed, this funding is predominantly used for allied health services, such as podiatry [29]. Furthermore, there is no current funding mechanism for remote patient monitoring or the delivery of a digital health program through Medicare [51]. As such, alternative forms of funding are likely to be required. Funding through PHN initiatives or private health insurance may provide pathways for sustainable reimbursement. State-level funding may also be viable, with precedent from the South Australian government supporting the sustained delivery of a virtual clinical care model [52]. Sustainable delivery of CAPS will require investment in training, technical infrastructure (eg, servers, product updates, and cybersecurity), and secure integration with primary practices. Scalability of the program will also require addressing interoperability barriers across diverse practice management systems, ensuring workforce availability and readiness, and improving digital health literacy among both clinicians and patients [53]. Demonstration of downstream cost benefits from program delivery (eg, reduced hospitalization) will also be essential to attract continued funding.

Identifying successful digital programs, which have been uplifted into a health service, can highlight factors that facilitate implementation. However, there is limited reporting on this process, and many programs face large implementation challenges [54]. A successful Australian example is Cardihab (Cardihab Pty Ltd) [55], a cardiac rehabilitation program offered via a smartphone app and clinician-facing portal. The program was evaluated in an RCT for effectiveness before implementation [56], and it is now offered to patients in multiple Australian states through private health insurance and certain private and public health services [55]. Medly, delivered in Canada, is another example of a mobile phone–based telemonitoring program for heart failure. Developed through a user-centered design process, the program was evaluated for effectiveness in an RCT [57,58]. Medly is currently offered through 9 health services throughout Toronto, supported by the University Health Network and Boehringer Ingelheim Canada [59]. The successful implementation of these digital programs indicates the importance of conducting an RCT in order to progress through the implementation pipeline. Data from this study will inform the future design of an RCT, including modification of CAPS technology and the model of care to be evaluated.

The strengths of our study included the diverse range of clinicians and wider service providers with expertise across the continuum of care, including those involved in acute, rehabilitation, and primary care. Given their mention by GPs during the interviews, practice nurses and practice managers could have been interviewed to understand their perceptions of adopting CAPS into primary care. However, we feel that our pragmatic convenience sample of content experts provided sufficient information power related to the richness and relevance of the data [25]. We used standardized procedures to demonstrate the program components and capabilities, with clinicians asked a similar set of questions to enhance dependability and consistency while still permitting flexibility to explore their views and experiences. Analysis of the data was undertaken by researchers independently with collaborative cross-checking and oversight of a third researcher in the second group. We demonstrated the capabilities of the program for patient end users, including self-monitoring and tailored support for goal attainment. However, we did not have the opportunity to involve clinicians in using and delivering CAPS to demonstrate enhanced management of actual patients. This means their perceptions and interpretations were purely conceptual, based on the demonstration provided during the focus groups or interviews. As such, a number of clinicians made assumptions and provided comments about a program they were not fully familiar with. The current lack of interoperability with practice management software or electronic health records makes integration of CAPS challenging. The current program design, which relies on regular review and response to patient-entered data, may impact clinical workflows and workload. Furthermore, the use of CAPS requires some level of digital literacy and the ownership of compatible devices among patients, as well as digital competence among clinicians, which may limit equitable use and adoption. Interviews for the first and second groups were conducted by different researchers; however, a consistent semistructured interview guide was used across all interviews, and the facilitators were experienced. Data analysis was also conducted by the same 2 researchers, and all the other researchers were involved in the discussion of the results.

The data collected regarding clinician perceptions will be integrated with the prior patient feedback collected [17]. This will be used to guide amendments to the program components (both patient and clinician facing; eg, patient traffic-light summaries) and updates of the technology. Their feedback will also guide the future model of care to be evaluated in a mixed-methods RCT, where patient participants will be recruited earlier in their recovery as recommended, and clinicians will be involved in the delivery of the program. We will also collect information to facilitate an economic evaluation of the program, a critical component of future implementation.

### Conclusion

Clinician perspectives regarding the use of CAPS for improving the secondary prevention of stroke indicate the potential of the program to address gaps in care. Participant feedback indicated a need to consider a viable and sustainable model of care that includes primary care without detrimentally impacting associated workloads. Further evaluations of the program regarding effectiveness in phase II and III RCTs are warranted.

## Supplementary material

10.2196/72873 Multimedia Appendix 1 Outline of semistructured stimulus questions.

10.2196/72873 Checklist 1 COREQ checklist.

## Abbreviations

CAPS: Care Assistant and support Program for people after Stroke or transient ischemic attack
CDM: chronic disease management
CSIRO: Commonwealth Scientific and Industrial Research Organization
GP: general practitioner
PHN: primary health care network
RCT: randomized controlled trial
TIA: transient ischemic attack
