# Supplementary material for: Clinician Perceptions of a Novel Multicomponent Digital Care Assistant and Support Program for People After Stroke or Transient Ischemic Attack (CAPS) for the Secondary Prevention of Stroke: Qualitative Study
Source: JMIR Hum Factors. 2025 Oct 9;12:e72873. doi: 10.2196/72873 (PMC12510440; doi:10.2196/72873)
Supplement: Multimedia Appendix 1 [file humanfactors-v12-e72873-s001.pdf]

Multimedia Appendix 2. Outline of semi-structured stimulus questions.

<table>
<tr><th>Topic</th><th>Phase I</th><th>Phase II</th></tr>
<tr><td>Experience & Usability</td><td colspan="2">How was navigating the user interface? What did you like/dislike?
How frequently would you review the health data collected?
What information did you find most useful?</td></tr>
<tr><td>Perceived benefits</td><td colspan="2">Could CAPS help patients living with stroke/TIA? Who would most benefit?</td></tr>
<tr><td rowspan="4">Implementation</td><td colspan="2">Where, by whom, and when should the program be delivered?
What challenges do you see patients facing with the program?
What data would you want to be alerted about?</td></tr>
<tr><td>-</td><td>Would you use CAPS in clinical practice? If so, how?</td></tr>
<tr><td></td><td>Are your patients using any form of digital health?</td></tr>
<tr><td>-</td><td>What challenges are there to clinician engagement?</td></tr>
<tr><td rowspan="2">Other</td><td colspan="2">How should a clinician be alerted (e.g., email)?
What changes would you suggest to the portal or app?</td></tr>
<tr><td>What other alerts should be developed?</td><td>-</td></tr>
</table>
